# Novel therapeutic targets, including IGFBP3, of umbilical cord mesenchymal stem-cell-conditioned medium in intrauterine adhesion

**DOI:** 10.1242/bio.060141

**Published:** 2024-02-12

**Authors:** Yuan Zhu, Mingjie Bao, Ting Wang, Xiaoyan Ai, Dewen Qiu, Changhua Wang

**Affiliations:** ^1^ Department of Gynecology, Jiangxi Maternal and Child Health Hospital, Nanchang, 330000, China; ^2^ Department of Obstetrics and Gynecology, Jiangxi Provincial People's Hospital, The First Affiliated Hospital of Nanchang Medical College, Nanchang, 330000, China; ^3^ Clinical laboratory, Jiangxi Maternal and Child Health Hospital, Nanchang, 330000, China

**Keywords:** Intrauterine adhesions, Umbilical cord mesenchymal stem cells, Conditioned medium, High-throughput qPCR, Endothelial fibroblasts

## Abstract

Mesenchymal stem cells play important roles in repairing injured endometrium. However, the molecular targets and potential mechanism of the endometrial recipient cells for stem cell therapy in intrauterine adhesion (IUA) are poorly understood. In this study, umbilical cord mesenchymal stem-cell-conditioned medium (UCMSCs-CM) produced positive effects on a Transforming growth factor beta (TGF-β) induced IUA cell model. RNA-sequencing was performed on clinical IUA tissues, and the top 40 upregulated and top 20 downregulated mRNAs were selected and verified using high-throughput (HT) qPCR in both tissues and cell models. Based on a bioinformatic analysis of RNA-sequencing and HT-qPCR results, 11 mRNAs were uncovered to be the intervention targets of UCMSCs-CM on IUA endometrium cell models. Among them, IGFBP3 was striking as a key pathogenic gene and a potential diagnostic marker of IUA, which exhibited the area under the curve (AUC), sensitivity, specificity were 0.924, 93.1% and 80.6%, respectively in 60 endometrial tissues. The silencing of IGFBP3 exerted positive effects on the IUA cell model through partially upregulating MMP1 and KLF2. In conclusion, RNA-sequencing combined with HT qPCR based on clinical tissues and IUA cell models were used in IUA research and our results may provide some scientific ideas for the diagnosis and treatment of IUA.

## Teaser

**Summary:** Eleven mRNAs were discovered to be the underlining intervention targets of UCMSCs-CM on intrauterine adhesion, which are of great significance for potential diagnosis and treatment.

## INTRODUCTION

Intrauterine adhesion (IUA) usually refers to endometrial repair disorders caused by uterine injury. Currently, there is no effective approach for the treatment of severe IUA because of failure in promoting endometrial regeneration, thus developing new methods to solve the problem may treat severe IUA effectively.

The main pathological changes in IUAs are inflammation and extracellular matrix accumulation, which in turn lead to endometrial fibrosis. Transforming growth factor beta 1 (TGF-β1) is recognized as a central profibrotic factor and the most powerful mediator promoting the epithelial–mesenchymal transition (EMT) (Feng et al., 2020). High expression of TGF-β1 has been demonstrated in both clinical IUA samples and animal IUA models (Abudukeyoumu et al., 2020). Currently, TGF-β1 is frequently used to induce IUA cell models with human endometrial stromal cells (HESCs) (Liu et al., 2020; Xie et al., 2020; Zhu et al., 2019).

Transplanting stem cells as a cell-based therapy to regenerate the endometrium could be a promising strategy for IUA treatment (Song et al., 2021; Yao et al., 2019). Transplanted stem cells provide morphological and functional benefits through multiple mechanisms including trophic support, cell replacement, regeneration of endogenous cells, immunosuppression and anti-inflammation. (Cervello et al., 2015; Du et al., 2012; Wang et al., 2016; Zhang et al., 2016).

Umbilical cord mesenchymal stem cells (UCMSCs) are considered to be good clinical tools and a promising approach for IUA treatment (Song et al., 2021; Xin et al., 2019). Conditioned medium (CM) is the culture medium containing the secretome of the stem cells. Application of CMs derived from MSCs has similar therapeutic effects in various kinds of disease models (Wang et al., 2021b). So far, however, few research papers on the use of UCMSCs–CM for IUA intervention have been published.

Some research concerning the positive effects of human UCMSCs on IUA has studied the underlining mechanisms from the perspective of stem cells (Ebrahim et al., 2018; Sun et al., 2021; Wang et al., 2021a). However, few studies have focused on the targets of recipient endometrial cells that respond positively to stem cell intervention, which we thought should be more practically significant for the development of new diagnostic and treatment methods for IUA.

## RESULTS

### CM of UCMSCs produced positive effects on the TGF-β1-induced IUA cell model

**Fig. 1.**
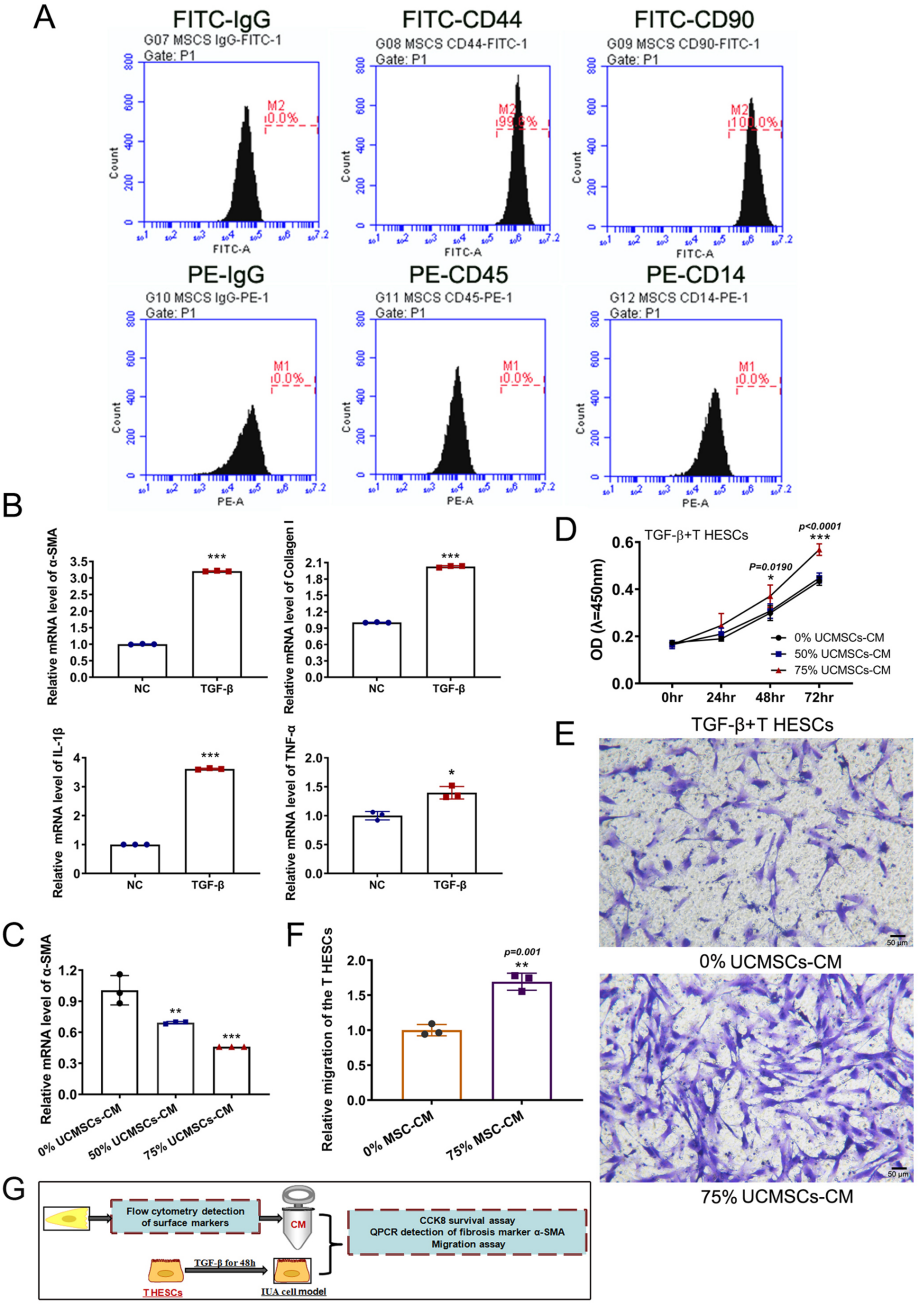
**UCMSCs-CM exerted positive effects on a TGF-β1-induced IUA cell model.** (A) Flow cytometric detection of surface markers of mesenchymal stem cells, FITC-CD44 and CD90 were used as positive markers and PE-CD45 and CD14 were used as negative markers. (B) The relative mRNA levels of fibrosis markers (α-SMA, Collagen-I) and inflammatory factors (IL-1β, TNF-α) were detected using qPCR, the measurement data are presented as the means±s.d., *n*=3; a two-tailed, independent sample *t*-test was carried out for the comparison of two conditions. **P<0.01, ***P<0.001 versus NC group. (C) The relative mRNA level of fibrosis marker α-SMA was detected using qPCR, the measurement data are presented as the means±s.d., *n*=3; ANOVA with a Bonferroni *post-hoc* test was used for multiple comparisons,**P<0.01, ***P<0.001 versus non-intervention group (0% UCMSCs-CM). (D) The CCK-8 assay of IUA model cells with or without UCMSCs-CM intervention, the measurement data are presented as the means±s.d., *n*=5; ANOVA with a Bonferroni *post-hoc* test was used for multiple comparisons,*P<0.05, ***P<0.001 versus non-intervention group. (E) Migration assay in transwell system to detect viability and motility of IUA cell models with or without UCMSCs-CM intervention (F) and the measurement data are presented as the means±s.d., *n*=3; *t*-test was carried out for the comparison of two conditions,**P<0.01 versus non-intervention group (0% UCMSCs-CM). (G) Overview of model grouping and experiments in this section.

Obtaining qualified mesenchymal stem cells is one of the prerequisites for this study. Firstly, the specific surface antigens of umbilical-cord-derived MSCs were detected by flow cytometry to confirm the quality of UCMSCs. The peak values of the UCMSCs-specific positive surface antigens CD44 and CD90 were nearly 100% and shifted to the right significantly, while the values of the negative markers CD45 and CD14 were 0%, proving that the UCMSCs were qualified (Fig. 1A). Subsequently, the relative messenger ribonucleic acid (mRNA) levels of fibrosis markers (alpha smooth muscle actin, α-SMA and Collagen-I) and inflammatory factors (Interleukin-1 beta, IL-1β, and tumor necrosis factor alpha, TNF-α) were used to evaluate the success of the IUA cell model. The real-time polymerase chain reaction (qPCR) results indicated that when T HESCs were induced by TGF-β1, the relative mRNA levels of α-SMA, Collagen-I, IL-1β, and TNF-α were all enhanced significantly, suggesting the successful construction of the IUA cell model (Fig. 1B).

Next, the qPCR assay evaluated the remission effect of 50% and 75% UCMSCs-CM on IUA cells. The relative mRNA level of α-SMA was obviously reduced in 50% and 75% CM groups when compared with the 0% CM group. In addition, the 75% CM intervention group displayed a better remission level than the 50% CM group (Fig. 1C). The Cell Counting Kit-8 (CCK-8) assay demonstrated that IUA model cells incubated with 75% UCMSCs-CM displayed a significant survival advantage over the non-intervention group (0% UCMSCs-CM), (Fig. 1D). Therefore, 75% UCMSCs-CM was used in subsequent experiments. The migration assay indicated when IUA model cells were incubated with 75% UCMSCs-CM for 48 h, the number of cells passing through the intermediate membrane in the transwell system increased markedly, suggesting a significant improvement in cell viability and motility (Fig. 1E,F). These results demonstrated that UCMSCs-CM exerted positive effects on TGF-β1-induced IUA model cells.

### RNA-sequencing of IUA samples collected clinically

**Fig. 2.**
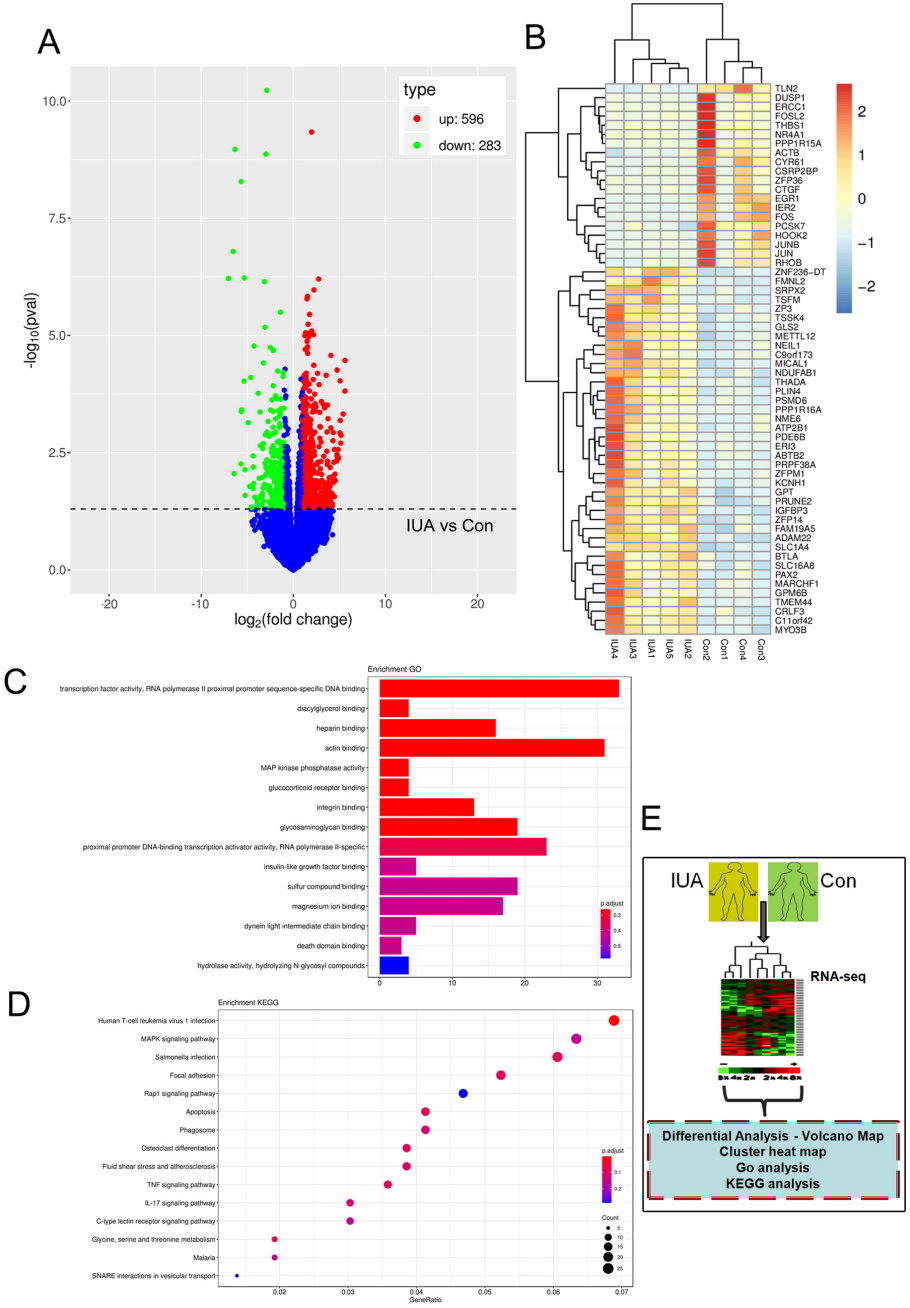
**RNA-sequencing of IUA samples collected clinically.** To reveal the differential transcriptome profile of the IUA group (*n*=5) versus the normal group (control, *n*=4), RNA-sequencing was implemented. (A) Volcanic map showing that 596 mRNAs were significantly upregulated and 283 mRNAs were significantly downregulated (IUA versus control, *P*<0.05, *|log2FoldChange|*>1.0). (B) Cluster map for the top 40 upregulated and the top 20 downregulated mRNAs. (C) GO and (D) KEGG enrichment analysis results of DEGs measured by bioinformatics analysis after RNA sequencing. (E) Overview of model grouping and experiments in this section.

The clinical IUA tissues could be more comprehensive and practical to reflect the differentially expressed gene (DEG) profiles under IUA status than any IUA cell models. To explore the possible DEGs in IUA, RNA-sequencing was performed using clinical IUA tissues and nine sequencing results were analyzed (IUA, *n*=5; control, *n*=4; one case of control tissue failed to enter the bioinformatics analysis phase due to the sequencing raw data being less than 6G). Through bioinformatic analyses, 596 significantly upregulated mRNAs and 283 significantly downregulated mRNAs were obtained (IUA versus control, *P*<0.05, |log2FoldChange| >1.0) (Fig. 2A). Subsequently, a cluster analysis was performed on the top 40 upregulated and top 20 downregulated mRNAs with a typical difference between the IUA and control groups according to the expression change multiple. Clusters were then annotated and clarified (Fig. 2B). Gene Ontology (GO) and Kyoto Encyclopedia of Genes and Genomes (KEGG) enrichment analyses were also performed. The top three enrichment cell functions revealed through GO analysis were: transcription factor, RNA polymerase II proximal promoter sequence-specific DNA binding, diacylglycerol binding and heparin binding (Fig. 2C). Meanwhile, the top three enrichment signaling pathways (according to counts) revealed through KEGG analysis were: human T-cell leukemia virus 1 infection, MAPK signaling pathway and salmonella infection (Fig. 2D).

### High-throughput qPCR validation after sequencing of clinical samples and cell model samples

**Fig. 3.**
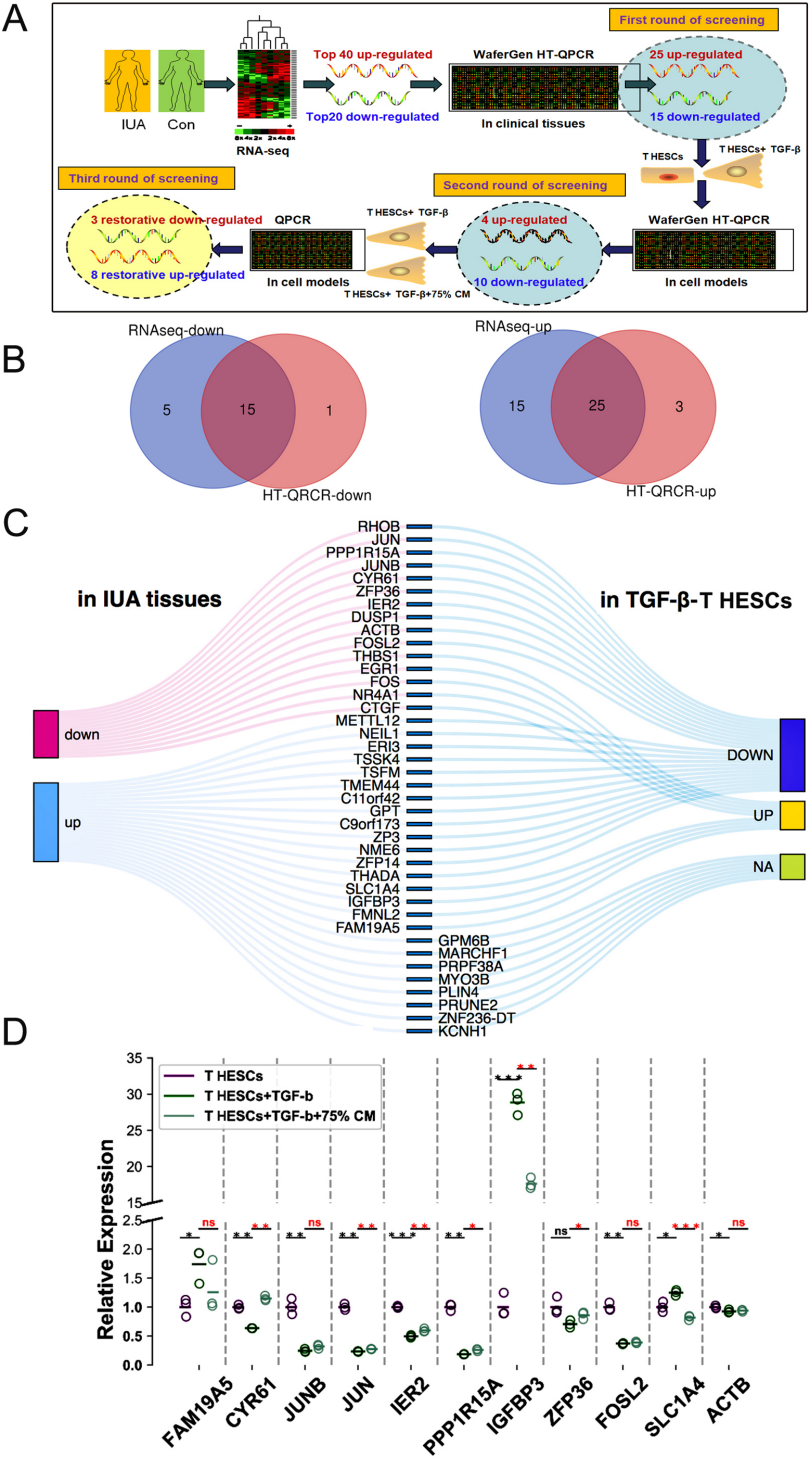
**HT-qPCR validation after sequencing of clinical samples and cell model samples.** (A) Schematic protocol of joint analysis on HT-qPCR verification of clinical samples and cell models with RNA-sequence. (B) The top 40 upregulated and top 20 downregulated mRNAs (IUA versus control) were verified with the WaferGen HT-qPCR system in 16 tissue samples (IUA: 8, control: 8), the intersection pie charts were drawn to show the qPCR verification results, the blue section represents the RNA-sequencing results and the red section represents the qPCR verification results in tissue samples. (C) Overview map of qPCR secondary validation of mRNAs in clinical tissues (IUA versus control, *n*=8 each) and two cell-model samples (T HESCs+ TGF-β versus T HESCs). (D) The relative mRNA levels of the potential intervention targets of UCMSCs-CM in three cell models detected by WaferGen HT-qPCR system (T HESCs, T HESCs+ TGF-β and T HESCs+ TGF-β+UCMSCs-CM). Note that there is a gap on the y-axis, which is inserted to reduce blank space. Independent sample *t*-test was used for inter-group comparison, *P<0.05, **P<0.01, ***P<0.001 between indicated groups.

Sequencing results often need to be validated by qPCR to eliminate false positive signals as much as possible, due to the DEGs generated by RNA-sequencing coming from clinical IUA tissues, but our sample size was very limited. The TGF-β-induced human endometrial stromal cell model is the most widely used IUA cell model for studying molecular mechanisms of IUA. Thus, high-throughput (HT)-qPCR verification was performed not only on clinical tissues but also on our IUA cell models to achieve dual insurance. The joint analysis and screening strategy was divided into three rounds (Fig. 3A). According to the RNA-sequencing of clinical samples, the top 40 upregulated and top 20 downregulated mRNAs (IUA versus control) were selected. Next, the 60 selected mRNAs were verified with the HT-qPCR in 16 tissue samples (IUA: 8, control: 8) (Fig. S1). Twenty-five overlapping mRNAs among the top 40 upregulated mRNAs and 15 among the top 20 downregulated mRNAs were verified by HT-qPCR in 16 tissues (IUA: 8, control: 8) (Fig. 3A,B), thus we obtained 40 follow-up mRNAs. Next, the 40 overlapping mRNAs were verified again with the HT-qPCR in IUA cell models. Accordingly, of the 25 enhanced mRNAs, only four were reconfirmed, and of the 15 downregulated mRNAs verified by qPCR in tissues, 10 were reconfirmed *in vitro* (T HESCs+TGF-β versus T HESCs) (Fig. 3C). After the second round of screening, the number of mRNAs was reduced to 14. Finally, to find potential intervention targets, we investigated which of the remaining 14 mRNA levels were changed in IUA cell models (T HESCs+TGF-β versus T HESCs) but restored after UCMSCs-CM intervention (T HESCs+TGF-β versus T HESCs+TGF-β+75% CM) by qPCR. The results indicated that 11 of the 14 candidates met our preset expression trend and were likely key targets for the positive intervention effect of UCMSCs-CM on IUA cells (Fig. 3D).

### Insulin-like growth factor-binding protein 3 (IGFBP3) could be a key pathogenic target of IUA, and IGFBP3 silencing exerted positive therapeutic effects on TGF-β-induced T HESCs

**Fig. 4.**
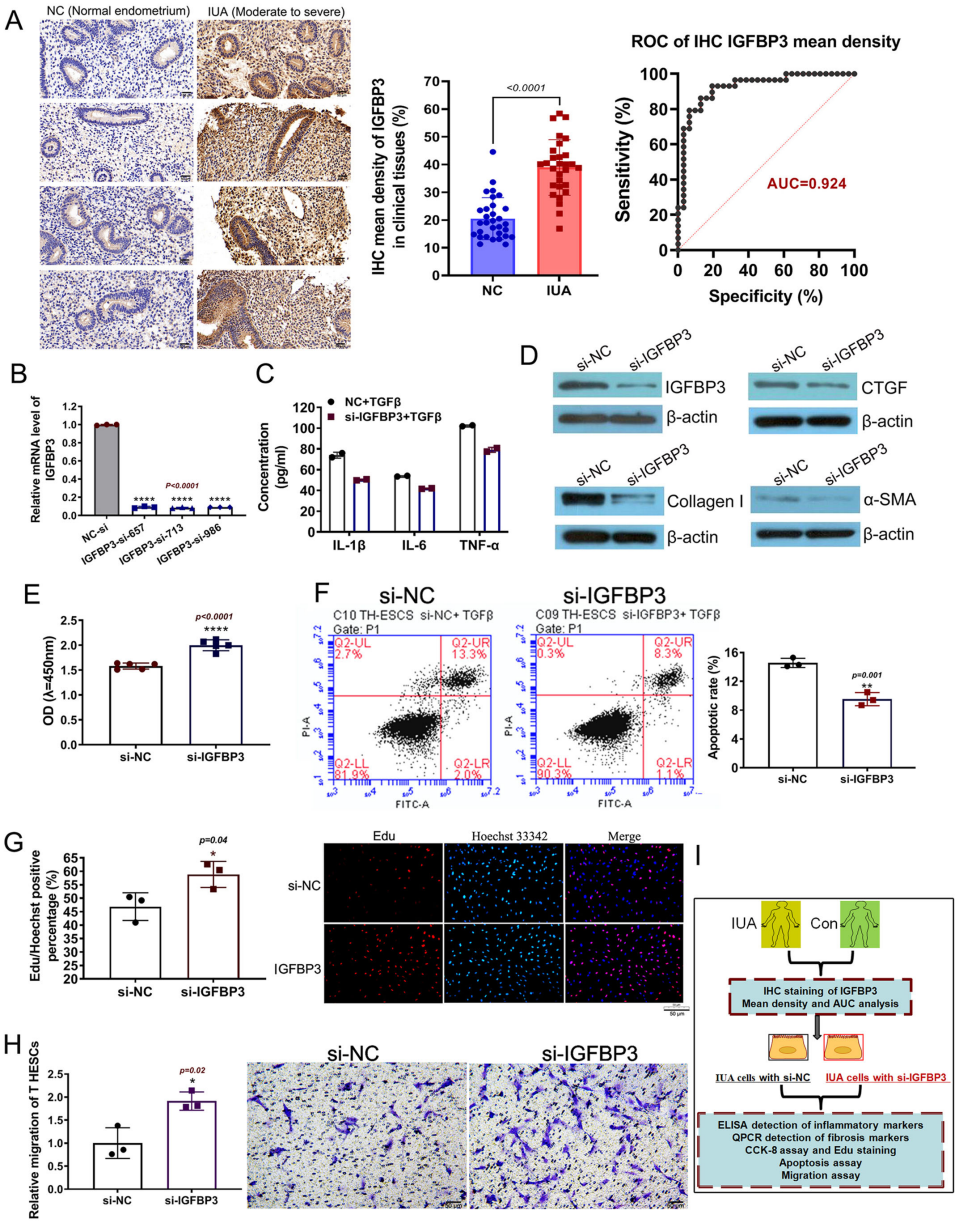
**IGFBP3 could be a key pathogenic target of IUA and IGFBP3 silence could exert positive therapeutic effects on TGF-β-induced T HESCs.** (A) Representative images of IHC staining of IGFBP3 on clinically collected IUA tissues (*n*=29) and normal endometrial tissues (*n*=31) (left). The magnification was 400×, scale bars: 50 μm. Statistical analysis of average density of IGFBP3 IHC (middle) and ROC curve (right). (B) qPCR verification of interference efficiency of different siRNA sequences to IGFBP3. The measurement data are presented as the means±s.d., *n*=3; ANOVA with a Bonferroni *post-hoc* test was used for multiple comparisons, ****P<0.0001 versus NC group (NC-si). (C) ELISA detection of related inflammatory factors. (D) The expression of IGFBP3 and fibrosis markers α-SMA, Collagen-I, CTGF were detected using western blotting in IUA cells with or without IGFBP3 KD. (E) The CCK-8 assay of IUA model cells with or without IGFBP3 silence, the measurement data are presented as the means±s.d., *n*=5; two-tailed, independent sample *t*-test was carried out for the comparison of two conditions, ****P<0.0001 versus NC group (si-NC). (F) Flow cytometric detection of apoptosis of IUA cell models with or without IGFBP3 KD based on annexin V-FITC, measurement data are presented as the means±s.d., *n*=3, two-tailed, independent sample *t*-test was carried out for the comparison of two conditions,**P<0.01 versus NC group (si-NC). (G) Edu staining was preformed to detect the proliferation of IUA model cells with or without IGFBP3 silence, the magnification was 100×, scale bar: 50 μm, the measurement data are presented as the means±s.d., *n*=3, two-tailed, independent sample *t*-test was carried out for the comparison of two conditions, *P<0.05 versus NC group (si-NC). (H) Migration assay in transwell system to detect viability and motility of IUA cell models with or without IGFBP3, the magnification was 100×, scale bars: 50 μm, measurement data are presented as the means±s.d., *n*=3; *t*-test was carried out for the comparison of two conditions,*P<0.05 versus NC group (si-NC). (I) Overview of model grouping and experiments in this section.

IGFBP3 was particularly eye catching, its mRNA level was upregulated nearly 28 times in IUA cell models compared to normal cells (T HESCs+TGF-β versus T HESCs). However, when the IUA cell model was treated with 75% UCMSCs-CM, the relative mRNA level of IGFBP3 was significantly decreased (Fig. 3D). The result suggested that IGFBP3 could not only be a pathogenic target of IUA but also one of the distinct targets of UCMSCs-CM in IUA models. Subsequently, IGFBP3 was detected using immunohistochemistry (IHC) staining in normal endometrial, moderate, and severe IUA tissues. The average optical density of IGFBP3 was significantly higher in IUA groups than in negative control cells (NC). The area under the curve (AUC) of IGFBP3 mean density in the sample was 0.924 after receiver operating characteristic (ROC) analysis, its average optical density threshold was >25.55, and the sensitivity and specificity were 93.1% and 80.6%, respectively. These results suggest that IGFBP3 may be one of the molecular indicators for IUA screening (Fig. 4A). To explore the gene function of IGFBP3, a loss-of-function model was constructed using siRNA silencing in TGF-β-treated T HESCs. Three specific siRNAs (si-657, si-713, and si-986; Table 2) have highly significant interference efficiency on IGFBP3 and were mixed equally in subsequent experiments (Fig. 4B).

Inflammatory infiltration and endometrial fibrosis are the two most common clinical features of IUA. Enzyme-linked immunosorbent assay (ELISA) detection showed IL-1β, IL-6, and TNF-α levels were all decreased in IGFBP3-knockdown (KD) IUA cells (Fig. 4C; si-IGFBP3+TGF-β versus si-NC+TGF-β). Next, a western blot analysis showed that in IGFBP3-KD IUA cells, α-SMA, Collagen-I, and connective tissue growth factor (CTGF) were all reduced when compared with IUA cells without IGFBP3 KD (Fig. 4D).

Higher cell proliferation, survival, migration, and lower cell apoptosis suggest a healthier cell state of T HESCs. CCK-8 assay indicated that the viability of IGFBP3-KD cells (si-IGFBP3) was significantly enhanced when compared with NC IUA cells (si-NC) (Fig. 4E). The annexin-V apoptosis assay indicated that the apoptosis rate of IUA cells with and without IGFBP3 KD was 9.4% and 15.3%, respectively, suggesting that IGFBP3 silencing could inhibit the apoptosis of IUA cells (Fig. 4F). Additionally, EdU staining was performed to evaluate cell proliferation, and the relative results indicate that IGFBP3 KD in IUA cells display a higher proportion of living cells with red EdU fluorescence signal (versus si-NC, Fig. 4G). The migration assay indicated that the number of IUA cells with IGFBP3 KD passing through the intermediate membrane in the transwell system (purple) was higher than in the NC IUA cells (si-NC), suggesting a significant improvement in cell viability and motility (Fig. 4H). These results demonstrate that IGFBP3 KD could exert positive therapeutic effects on TGF-β-induced T HESCs.

### IGFBP3 overexpression reduced the positive effect of UCMSCs-CM on IUA cell models to a certain extent

**Fig. 5.**
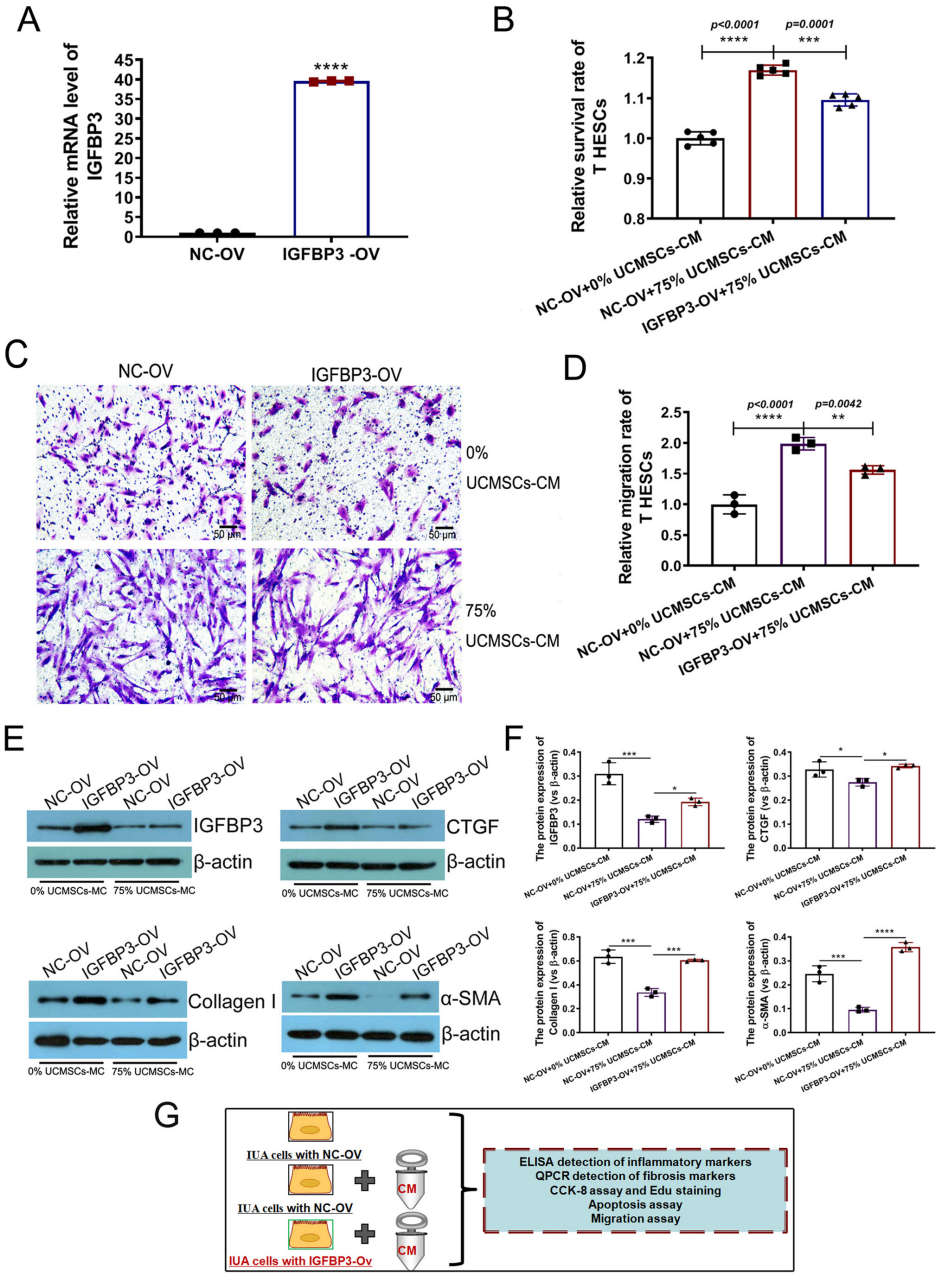
**IGFBP3 overexpression diminished the positive effect of UCMSCs-CM on IUA cell models to a certain extent.** (A) qPCR was used to confirm the successful construction of IGFBP3-overexpression model, the measurement data are presented as the means*±*s.d.*, n*=3*,* two-tailed, independent sample *t*-test was carried out for the comparison of two conditions,****P<0.0001 versus NC group (NC-OV). (B) The relative survival rate of IGFBP3 overexpressed IUA model cells with or without UCMSCs-CM treatment were detected using the CCK-8 assay, the data are presented as the means±s.d., *n*=5; ANOVA with a Bonferroni *post-hoc* test was used for multiple comparisons,***P<0.001, ****P<0.0001 between indicated groups. (C,D) A migration assay in a transwell system was performed to detect viability and motility of IGFBP3-overexpressed IUA cell models with or without UCMSCs-CM treatment. The magnification was 100× and the measurement data are presented as the means±s.d., *n*=3; ANOVA with a Bonferroni *post-hoc* test was used for multiple comparisons,**P<0.01, ****P<0.0001 between indicated groups. (E) The expression of IGFBP3 and fibrosis markers α-SMA, Collagen-I, CTGF were detected using western blots in IGFBP3-overexpressed cell models with or without UCMSCs-CM treatment and the gray data of each group were statistically analyzed (F), the measurement data are presented as the means±s.d., *n*=3; ANOVA with a Bonferroni *post-hoc* test was used for multiple comparisons,*P<0.01, ***P<0.001, ****P<0.0001 between indicated groups. (G) Overview of model grouping and experiments in this section.

To further prove that IGFBP3 is one of the key targets for UCMSCs-CM exerting positive effects on IUA cell models, an IGFBP3 gain-of-function cell model (IGFBP3-overexpression) was established and verified by qPCR (Fig. 5A) and western blotting (Fig. 5E, left upper lanes 1 and 2). Through the CCK-8 assay, compared with the negative control IUA cells (NC-overexpression, OV), the pro-survival effect of 75% UCMSCs-CM was observed to be significantly weakened in IUA cells with IGFBP3 overexpression (IGFBP3-OV) (Fig. 5B). The migration assay indicated that IGFBP3 overexpression reduced the number of IUA cells passing through the intermediate membrane of the transwell system without UCMSCs-CM treatment. When UCMSCs-CM was added for intervention, the pro-motility effect of UCMSCs-CM was also alleviated in IUA cells overexpressing IGFBP3 (Fig. 5C,D). These results suggest that one way that UCMSCs-CM positively affects IUA is by reducing IGFBP3 expression. The results of western blotting indicated that IGFBP3 overexpression significantly enhanced the expression of fibrosis marker proteins (versus NC-OV). After the intervention with UCMSCs-CM, fibrosis marker proteins' expressions decreased significantly due to the curative effect of UCMSCs-CM. However, the expression of fibrosis marker proteins in IGFBP3-overexpressing IUA cells (IGFBP3-OV+75% UCMSCs-CM) was still observably stronger than in control IUA cells (NC-OV+75% UCMSCs-CM) (Fig. 5E,F). These results suggest that the IGFBP3 overexpression weakened the positive effect of UCMSCs-CM on IUA cell models to a certain extent.

### UCMSCs-CM alleviates IUA by indirect regulation of MMP1, MMP10, and KLF2 through inhibiting IGFBP3

**Fig. 6.**
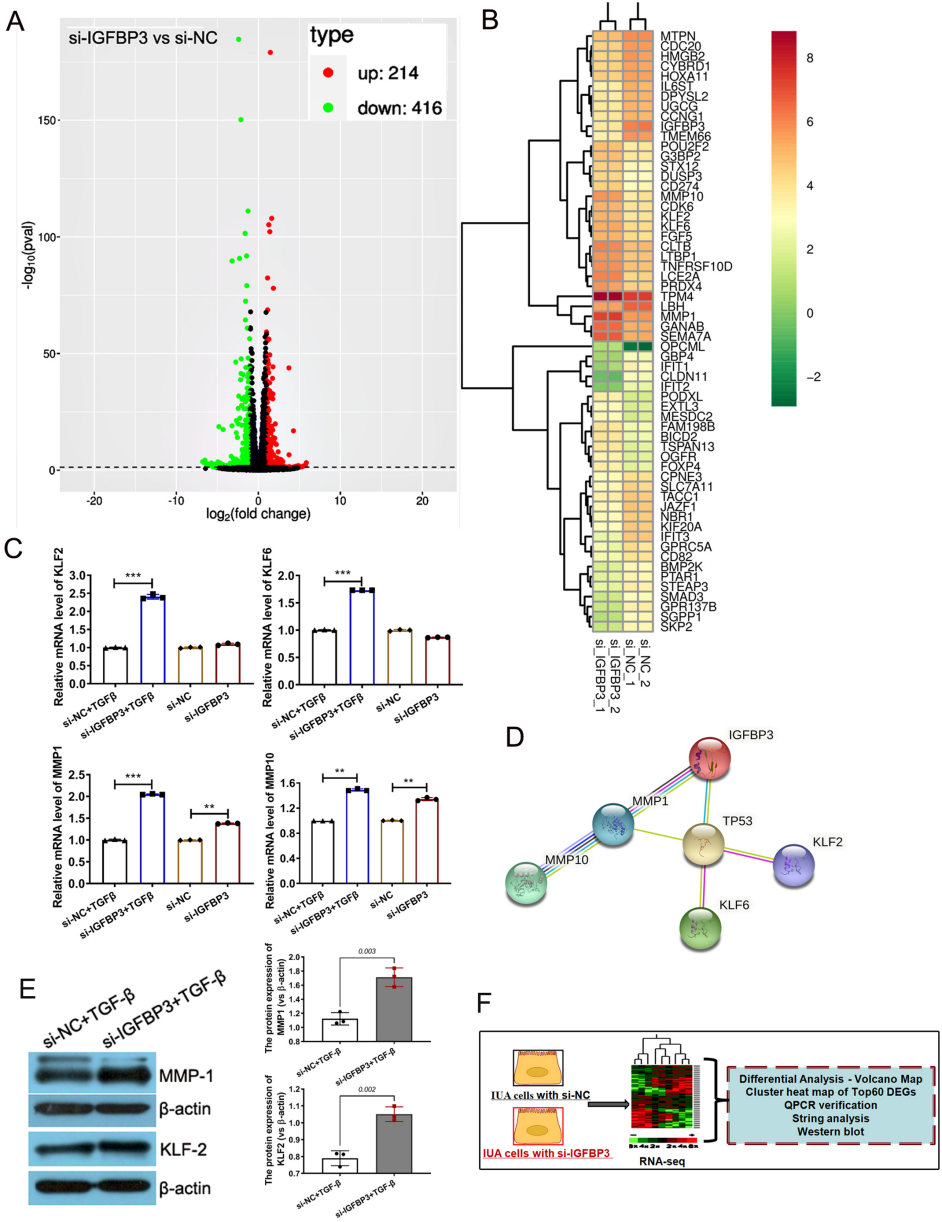
**UCMSCs-CM alleviates IUA by indirect regulation of MMP1, MMP10 and KLF2 through inhibiting IGFBP3.** (A) Volcanic map showing 214 upregulated mRNAs and 416 downregulated mRNAs between IGFBP3-silenced and relative NC group. (si-IGFBP3 versus si-NC, *P*<0.05, *|log2FoldChange|*>1.0). (B) Cluster map for the top 60 deregulated mRNA after IGFBP3 silencing. (C) qPCR was used to verify the downstream factors of interest MMP1, MMP10, KLF6 and KLF2 deduced from RNA-seq, the measurement data are presented as the means±s.d., *n*=3; ANOVA with a Bonferroni *post-hoc* test was used for multiple comparisons,**P<0.01, ***P<0.001 between indicated groups. (D) Protein correlation diagram drawn by string software. (E) Western blot verification of MMP1 and KLF2 expression of each group (left), and the gray data of each group were statistically analyzed (right), the measurement data are presented as the means±s.d., *n*=3; two-tailed, independent sample *t*-test was carried out for the comparison of two conditions,*P<0.01, ***P<0.001*,* between indicated groups. (F) Overview of model grouping and experiments in this section.

To explore the downstream pathways of IGFBP3 exerting its biological function, RNA-sequencing was performed in IUA cell models (TGF-β-treated T HESCs) with or without IGFBP3 KD (si-NC and si-IGFBP3, two parallel samples each). We obtained 214 significantly upregulated mRNAs and 416 significantly downregulated mRNAs (versus si-NC, *P*<0.05, |log2FoldChange| >1.0) (Fig. 6A). Subsequently, a cluster analysis was performed, and the top 60 deregulated mRNAs, according to the *P-*value between the two groups, were mapped and clarified (Fig. 6B). The expression of MMP1 and MMP10, which were reported to have the potential to limit fibrotic responses to injury, increased with IGFBP3 KD (Craig et al., 2015). In addition, Kruppel-like factor 2 (KLF2) levels, reported to be critical for antifibrosis by maintaining endothelial barrier integrity and preventing gap formations, were also increased with the IGFBP3 KD (Marrone et al., 2015; Rane et al., 2019). qPCR confirmed the mRNA levels of KLF2, KLF6, matrix metallopeptidases (MMP)-1, and MMP10 were all upregulated significantly in IGFBP3 loss-of-function IUA cell models (Fig. 6C). In addition, the online software String (https://cn.string-db.org/) was used to show the relationship between these proteins. On the one hand, the results indicated a strong correlation between IGFBP3 and MMP1, with a combined score of 0.949. Taking MMP1 as a bridge, IGFBP3 may regulate MMP10. On the other hand, IGFBP3 could not be directly associated with KLF2 or KLF6 unless the closely related protein tumor protein 53 (TP53) was involved (Fig. 6D). However, subsequent western blot validation indicated that only MMP1 and KLF2 expression significantly enhanced with the IGFBP3 KD (Fig. 6E). The above results suggest that UCMSCs-CM alleviates IUA by inhibiting IGFBP3 and causes corresponding expression alterations of its downstream targets, such as MMP1 and KLF2, thus exerting positive therapeutic effects on TGF-β-induced T HESCs.

## DISCUSSION

Bone-marrow-derived MSCs (Ventura Ferreira et al., 2018), human endometrial side population (Masuda et al., 2015), adipose MSCs (Marofi et al., 2017), endometrial MSCs (Deane et al., 2013), human embryonic stem cells (Azizi et al., 2018), human UCMSCs (Wang et al., 2021a), and human amniotic mesenchymal stromal cells (Gan et al., 2017) were reported to be involved in the field of IUA treatment research. Human UCMSCs were the best clinical choice due to their easy availability and low immunogenicity. In addition, studies have indicated that human UCMSCs have positive effects on IUA (Sun et al., 2021; Wang et al., 2021a; Zheng et al., 2020). Therefore, the present study decided to work with human UCMSCs rather than other types of stem cells.

Stem cell functions on IUA are mainly attributed to their paracrine mechanism (Yao et al., 2019). Evidence suggests that stem cell transplantation improves the local microenvironment in injured tissues by secreting various paracrine factors harvested in CM, which are advantageous for repairing and rejuvenating injured cells and tissues (Ling et al., 2019). Many studies have confirmed that direct intervention with stem cell CM but not exosomes, can have positive effects (Lin et al., 2018; Zhu et al., 2019). A previous study indicated that intrahepatic injection of adipose stem cell (ADSC)-CM was a new, cell-free method to improve hepatic ischemia-reperfusion injury (Zhang et al., 2022). The CM of menstrual-blood-derived MSCs from non-endometriosis patients could improve the symptoms of endometriosis (Sahraei et al., 2022). Although stem-cell-derived exosomes have attracted much attention, this study took the lead in focusing on the interventional efficacy of UCMSCs-CM on IUA.

In our research, the number of clinical tissues collected for RNA-sequencing was small, so we tried to find other public corresponding sequencing data from the Genomic Spatial Event (GSE) dataset and conduct conjoint analyses to increase the power of the sequencing results. Unfortunately, only one available dataset (GSE224093) was found to disclose RNA sequencing results of endometrial tissue from human IUA and NC groups. Moreover, since only the FPKM values were provided and it covered not only mRNAs but also non-coding RNAs, GSE224093 cannot be merged with our RNA-sequencing data. We could only check the 11 potential targets in the GSE224093 to roughly test the consistency. The results showed that out of the 11 mRNAs, eight were consistent, while three, FAM19A5, FOSL2, and SLC1A4, were inconsistent (Fig. S2, IUA versus NC). However, both clinical IUA tissue RNA-sequencing results of GSE224093 and our group were still based on a small number of samples, therefore, the subsequent research in the study focused on our own data.

We noticed that there was a striking lack of concordance in transcriptional responses when comparing between the in TGF-β induced T HESCs and the *in vivo* tissues. We speculated that since our clinical sample size was relatively small, and cells involved in the pathogenesis of IUA include not only human endometrial stromal cells but also human endometrial epithelial cells, human embryonic stem cells and macrophages, another reason for the low consistency between our IUA clinical tissues and cell models may be that a single-cell model could not fully reflect tissue-level transcriptional differences.

IGFBP3 is known for its pleiotropic ability to regulate cellular processes such as proliferation, apoptosis, and differentiation (Ushakov et al., 2021). A previous study indicated that the regulation of IGFBP3 by BMP2 has a role in human endometrial remodeling after embryo implantation, and the increased expression of IGFBP3 promoted MMP2 expression and cell migration (Luo et al., 2020). Our study found that in the IUA cell model, IGFBP3 loss-of-function reduced the secretion of inflammatory factors (Fig. 4C), weakened fibrosis marker expression (Fig. 4D), and improved the viability and motility of IUA cell models (Fig. 4E–H). The difference between our study and the earlier one lies first in the different pathophysiological processes. Secondly, IGFBP3 was described as the downstream factor of Luo's research protagonist BMP2, while it was the research target screened by multiple bioinformatics analyses in our study. In addition, the biological function of IGFBP3 in IUA cell models was explored in more detail in our research; in previous studies, MMP2 was found to be a downstream factor of IGFBP3 (Ushakov et al., 2021). Through the RNA-sequencing of IGFBP3-silenced cell models, we did not find the corresponding expression change of MMP2, but MMP1 and MMP10 were significantly upregulated. We tried to explore the relationships among IGFBP3, MMP1, MMP10, KLF2 and KLF6 proteins through string and observed a strong potential correlation between IGFBP3, MMP1, and MMP10. However, IGFBP3 could not be directly associated with KLF2 or KLF6 unless the closely related protein TP53 of IGFBP3 was involved. In addition, we failed to find the differential expression of TP53 in the RNA-sequencing results of the IGFBP3 loss-of-function cell model, which suggests other potential molecular regulatory mechanisms that have not been predicted take place.

As shown in Fig. 5B–D, reduced CM-associated properties in IGFBP3-OV cells (versus NC-OV+75% UCMSCs-CM). Meanwhile, there were still significant intergroup differences between IGFBP3-OV+ 75% UCMSCs-CM and NC-OV+0% UCMSCs-CM. We speculated that these results indicated IGFBP3 could not be the only target of CM in the IUA cell model, so even if IGFBP3 was overexpressed, there were still other important effector targets in the IUA model that respond to CM intervention.

This study did not implement corresponding experiments to support the experimental results *in vivo*, which will be conducted in our next experimental plan. However, based on a novel method of RNA-sequencing and HT-qPCR interactive bioinformatic analyses, this study still revealed that UCMSCs-CM positively affected TGF-β1-induced IUA cell models. The proposed 11 mRNAs could be not only the underlining intervention targets of UCMSCs-CM on IUA but also candidate targets for the development of non-invasive diagnostic methods for IUA based on menstrual blood, for example. IGFBP3 was one of them, which makes it a promising therapeutic target of IUA even without stem cell intervention.

## MATERIALS AND METHODS

### Cell culture and preparation of CM of umbilical cord mesenchymal stem cells

Human UCMSCs (passage 3) were purchased from Hunan Science & Well Biotechnology (Changsha, China) and cultured in StemGro MSC expansion medium (Basal Media, Shanghai, China) to 80% confluence. Then, the medium was replaced with serum-free Dulbecco's modified Eagle's medium (DMEM)/F12. After 48 h incubation, the CM was collected and centrifuged at 4500 ***g*** for 10 min. All the CM in this study was collected and used freshly without freezing.

Human endometrial fibroblasts (T HESCs) (CRL-4003), which were cultured normally in DMEM/F12 with 10% fetal bovine serum (FBS) and 1% Penicillin-Streptomycin Solution (P/S), were purchased from ATCC (with short tandem repeat, STR identification). The IUA cell model was constructed by treating T HESCs cells with 10 ng/ml TGF-β for 48 h (Liu et al., 2020; Zhu et al., 2019). The establishment of UCMSCs-CM intervention model was as follows: after TGF-β treatment, UCMSCs-CM was diluted with DMEM/F12 to 75% at 3:1 ratio. Then, T HESCs were incubated with UCMSCs-CM for 48 h (Zhu et al., 2019).

### Clinical specimens

IUAs (*n*=8, including 2 mild, 2 moderate and 4 severe) and normal control endometrial tissues (*n*=8) were collected from April to June in 2021 in Jiangxi Provincial Maternal and Child Health Hospital (Table S1). Control group: pathologically normal endometrial tissues from women aged 25–35 years with normal menstruation needing IUD placement for medical reasons. IUA group: moderate and severe intrauterine adhesions were diagnosed by hysteroscopy (gold standard) according to IUA diagnostic grading scoring standard of China. All volunteers were younger than 38 years old and signed informed consent, which was approved by the ethics committee of Jiangxi Maternal and Child Health Hospital (EC-KT202143). The IUA tissues were excised under the hysteroscopy by the annular electrode carefully, normal endometrium was carefully excised by medical curette. All clinical experiments were conducted in accordance with the principles of the declaration of Helsinki. The clinical specimens were frozen at −80°C after resection immediately and prepared for RNA-sequencing and qPCR.

### RNA sequencing

Ten tissues were selected for sequencing (IUA versus control, *n*=5 each). In IUA group, the tissues were clinically identified as moderate to severe (4 severe and 1 moderate). Following the manufacturer's recommendations, total RNAs were extracted from the clinically IUA and the control tissues by Invitrogen TRIzol Plus RNA extraction kit, and sequencing libraries were generated using NEBNext Ultra II RNA Library Prep Kit. PCR products were purified by AMPure XP system, library quality was determined by Agilent Bioanalyzer 2100 system, and then sequenced on an Illumina Novaseq platform 150 PE mode. All generated RNA-sequencing data were deposited into NCBI-SRA database (https://www.ncbi.nlm.nih.gov/sra/) under the project number of PRJNA 916532. Quality control of raw data and clean data was achieved using the FastQC. Paired-end clean reads were mapped to the GRCh37 genome by Hisat2. HT-Sequencing was used to quantify the reads numbers mapped to each gene. Differential expression analysis of IGFBP3 silenced and NC IUA model cells was performed using the DESeq2. The DEGs were selected by |log2FoldChange|>1.0 and significance *P*-value<0.05. GO and KEGG enrichment analysis was implemented by the clusterProfiler R package.

### HT and general qPCR

The RNAs from a total of 19 samples verified on the WaferGen included control and IUA samples collected clinically (*n*=8 each), the T HESCs NCs, T HESCs pathogenic model samples (TGF-β) and UCMSCs-CM intervention model samples (TGF-β+CM) (three cell-model samples). Total RNAs were extracted from the endometrial tissues and T HESCs using RNAsimple total RNA kit (catalogue number PD419, Tiangen, China) and then were reverse transcribed using the RevertAid First Strand cDNA Synthesis Kit (catalogue number K1622, ThermoFisher Scientific, MA, USA).

The post sequencing qPCR verification used the Wafergen smartchip HT qPCR system according to the manufacturer’s instructions. General qPCR was performed using SYBR Green Supermix (catalogue number FP205, Tiangen, China) in the Roche Light Cycler 480 system. Three independent experiments were carried out and the relative quantification comparative Ct method was used to quantify the relative mRNA levels of target genes. The primer sequences are listed in Table 1. The qPCR measurement data are presented as the means±standard deviation (SD) (*n*=3).

**Table 1.**
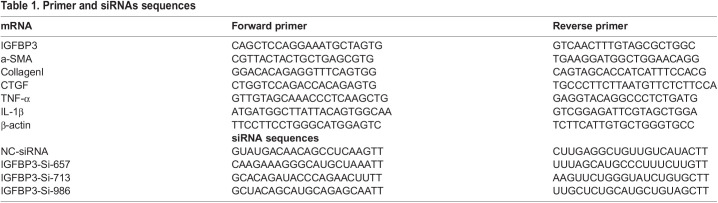
Primer and siRNAs sequences

### Cell survival assay

To assess the cell survival of T HESCs in different groups, the CCK-8 assay was performed as previously described (catalogue number K1018, APExBio, USA) (Li et al., 2019). T HESCs with different intervention strategies were cultured in 96-well culture plates (1×10^3^ cells per well). At predetermined time points, the medium was replaced with 100 μl Dulbecco's Modified Eagle's Medium (DMEM)/F12 containing 10 μl CCK-8 reagent. After 3 h incubation, the optical absorbance at 450 nm was measured.

### EdU incorporation assay

T HESCs were incubated with 10μM 5-Ethynyl-2′-deoxyuridine (EdU, ThermoFisher Scientific, catalogue number A10044,) for 4 h and then were fixed with 3.7% formaldehyde for 15 min. EdU incorporation was tested according to manufacturer's instructions. Images were taken on the confocal laser scanning microscope system (Nikon, Tokyo, Japan). Cells which were positive for both EdU and Hoechst 33342 (SelleckChem, USA, catalogue number S0485) were counted using Image J (Wayne Rasband, NIH) and were used to calculate the percentage of EdU-positive cells.

### Loss/gain-of-function cell models of IGFBP3

T HESCs were inoculated into a six-well plate (2 ml per well) with a density of 2.0×10^5^ cells/ml and incubated overnight in a 5% CO_2_ cell incubator at 37 °C. When the cells reached around 70–80% confluence, loss-of-function T HESCs model of IGFBP3 was achieved by transfecting specific siRNA sequences into T HESCs (final concentration was 100 nM, sequence information listed in Table 1) using 5µl lipofectin3000 (Invitrogen, USA); gain-of-function cell models of IGFBP3 were also achieved by transfecting 4 μg IGFBP3 overexpression synthetic plasmid into T HESCs using 5 μl lipofectin3000. After 48 h, the successful interference (si-IGFBP3) and overexpression (OV-IGFBP3) of IGFBP3 in T HESCs were confirmed by qPCR and western blotting.

### Western blotting

Western blotting was carried out as previously described (Ai et al., 2020). Cells in each group were lysed, protein concentrations were measured using a BCA Protein Assay Kit (ThermoFisher Scientific, catalogue number 23227). Equal amounts of protein were separated with SDS-PAGE and then blotted onto PVDF membranes (Millipore, MA, USA, catalogue number ISEQ00010). Next, the PVDF membranes were blocked in TBS containing 5% bovine serum albumin (BSA, Sigma-Aldrich, MO, USA, catalogue number A6003) and 0.05% Tween 20 for 1.5 h, and were further incubated at 4°C overnight using the relevant antibodies. The primary antibodies used were as follows: anti-IGFBP3 (Immunoway, Suzhou, China, catalogue number YT5518,), anti-α-SMA (Immunoway, catalogue number YM3364), anti-CollagenI (Abcam, USA, catalogue number ab90395), anti-CTGF (Immunoway, catalogue number YT6207), anti-β-actin (Immunoway, catalogue number Ym3028). The amount of the protein of interest was normalized to the densitometric units of β-actin.

### IHC

In order to check the diagnostic power of IGFBP3, a total of 60 additional paraffin sections provided by the biological sample bank of Jiangxi Maternal and Child Health Hospital were used (endometrial tissue sections from 29 moderate to severe IUA patients and 31 women with healthy endometrium, NC). The IHC staining was performed as previously described (Ai et al., 2020). The IGFBP3 antibody (Immunoway, catalogue number YT5518, 1:100) was added dropwise and at the end of the experiment, all slides were observed and photographed under panoramic scanning system (Pannorramic MIDI, Hungary). IHC mean density was measured using ImageJ software, the statistical analysis and ROC curve drawing were performed by GraphPad Prism 9.0 software.

### ELISA assay

The T HESCs cell culture supernatant was collected for ELISA detection of inflammatory factors after TGF-β treatment for 48 h with IL-β, IL-6, TNF-α ELISA kits according to the manufacturer's instructions (Lichen, Shanghai, China).

### Flow cytometry

For human UCMSC characterization, cells were harvested and incubated with labeled primary antibodies (PE-CD45: 368509, PE-CD14: 367103, FICT-CD44: 338803, and FICT-CD90: 328107, Biolegend, USA) at 4°C for 30 min. Then, the cells were washed with phosphate-buffered saline, and analyzed with a BD Accuri C6 (BD Biosciences, NJ, USA).

Flow cytometry was also used to detect cell apoptosis with annexin-V-FITC method (catalogue number 556547, BD Biosciences) according to the instructions. Cell Quest software (BD Biosciences) was used to analyze the result. The experiments were carried out three times in parallel.

### Migration assay

1.0×10^5^/ml T HESCs were inoculated into six-well culture plates (2 ml/well) and cultured at 37°C until about 80% confluence and then treated with TGF-β for 48 h. Cells were collected and diluted at 1.5×10^4^ per 100μl then added to the transwell upper chamber. Serum free medium or UCMSCs-CM (300 μl) was added to the upper chambers. 500 µl medium containing 20% FBS was added into the lower chambers and cultured in 37°C, 5% CO_2_ for 48 h. 4% tissue fixing solution was added to the transwell chambers and they were then fixed at room temperature for 20 min. Lastly, 0.1% Crystal violet was added for 15 min (Xu et al., 2021).

### Statistical analysis

All experimental results listed in this research are expressed as the mean±SD GraphPad Prism 9.0 software (GraphPad Software, San Diego, CA, USA) was used to perform the statistical analysis. Two-tailed, independent sample *t*-test was carried out for the comparison of two conditions. ANOVA with a Bonferroni's *post-hoc* test was used for multiple comparisons. The *P*-value <0.05 was considered statistically significant.

## Supplementary Material

10.1242/biolopen.060141_sup1 Supplementary information

Table S2. Western blot quantification
