# Supplementary material for: Novel therapeutic targets, including IGFBP3, of umbilical cord mesenchymal stem-cell-conditioned medium in intrauterine adhesion
Source: Biol Open. 2024 Feb 12;13(2):bio060141. doi: 10.1242/bio.060141 (PMC10886714; doi:10.1242/bio.060141)
Supplement: Supplementary information [file biolopen-13-060141-s1.pdf]

Biology Open (2024): doi:10.1242/bio.060141: Supplementary information

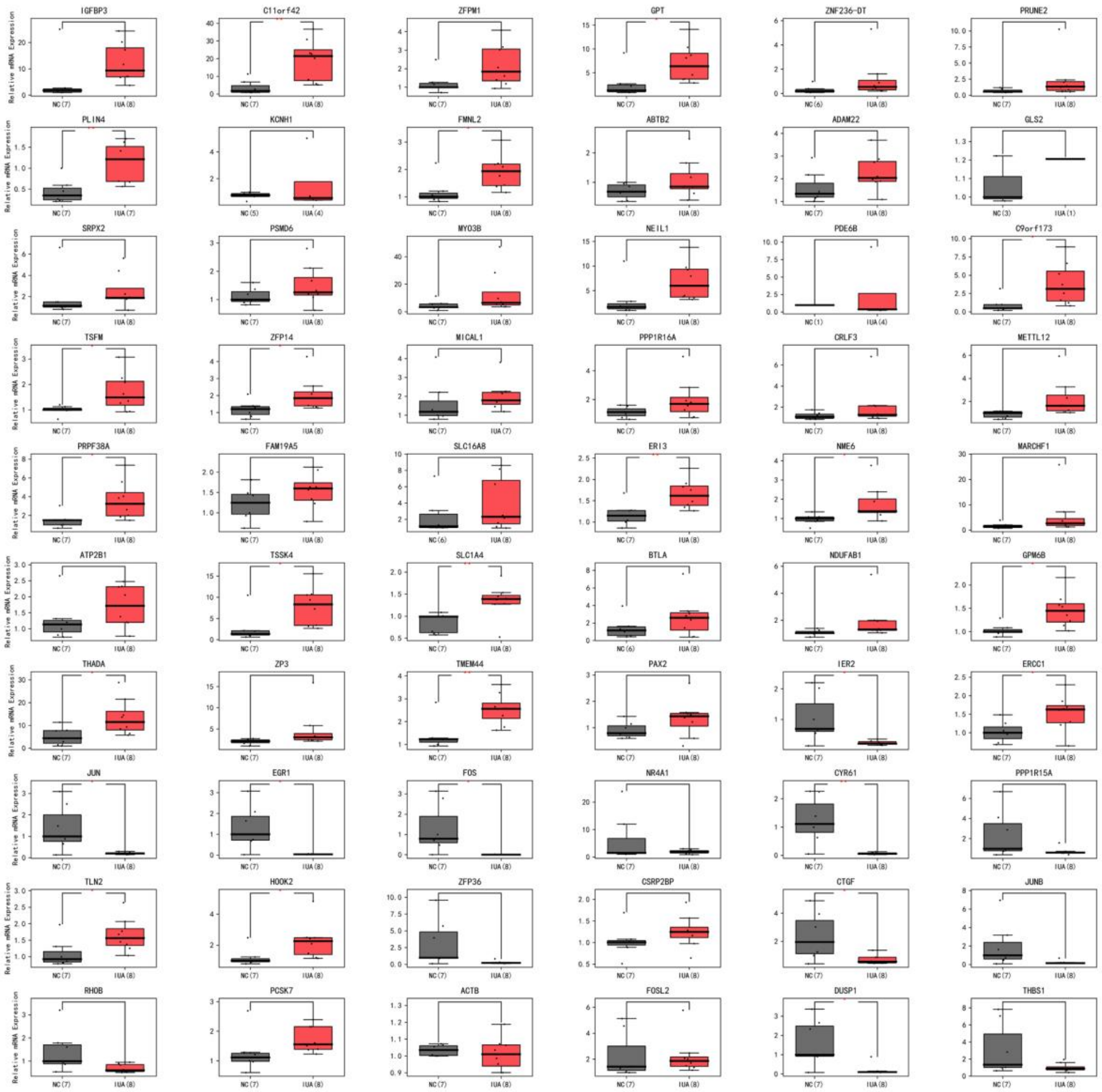

Fig. S1. High throughput QPCR validation after sequencing of clinical samples.

NC: control tissues,in gray; IUA : IUA tissues, in red. The left vertical axis represents the relative mRNA level (relative to the internal reference β-actin) the measurement data are presented as the means ± SD, n= 3; *P < 0.05, **P < 0.01 vs NC group.

Biology Open • Supplementary information

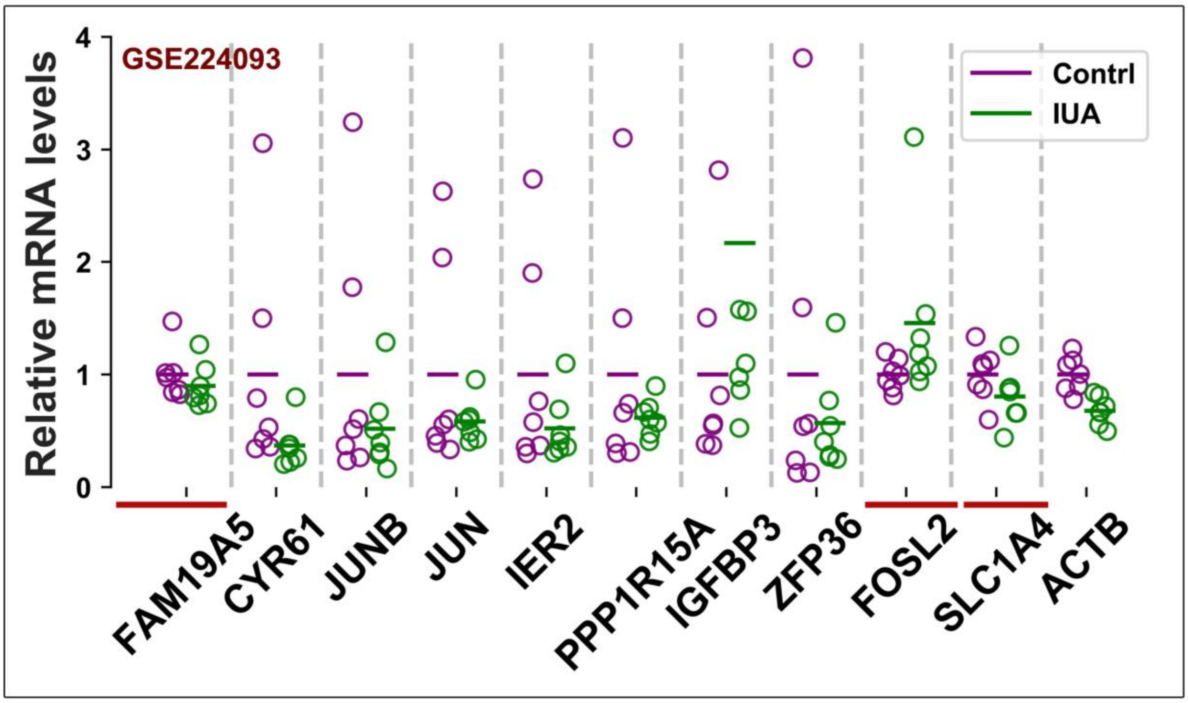

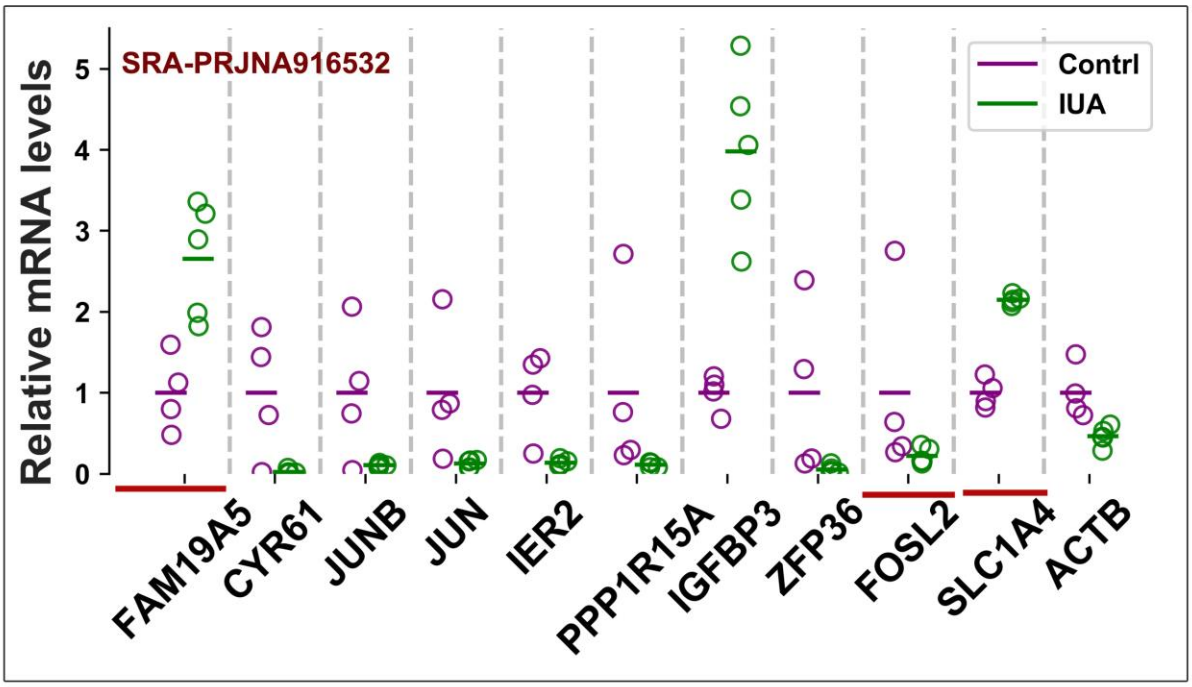

Fig. S2. 11 potential targets were checked again in the GSE224093 dataset.

Relative mRNA levels of 11 key targets based on FPKM values of RNA seq of clinical IUA tissues. The relative mRNA levels of the 11 potential intervention targets in clinical IUA tissues based on the FPKM values of GSE224093 (Control:7 cases, IUA: 7 cases) . (B) Relative mRNA levels of 11 potential intervention targets in clinical IUA tissues based on the FPKM values of RNA Seq in this study (SRA- PRJNA916532. Control:4 cases, IUA: 5 cases). The red lines below indicated mRNAs that were inconsistent between the two RNA-seq datasets

Biology Open • Supplementary information

Table S1. Clinical Specimen Information and usage

Clinical Specimen Information			
Sample	pathological condition	Sample usage	
		RNA-seq	HT-QPCR
IUA group			
1	Mild IUA	--	Yes
7	Moderate IUA	Yes	Yes
8	Mild IUA	--	Yes
9	Moderate IUA	--	Yes
10	Severe IUA	Yes	Yes
11	Severe IUA	Yes	Yes
15	Severe IUA	Yes	Yes
16	Severe IUA	Yes	Yes
Contrl group			
3		Yes	Yes
4		Yes	Yes
5		Yes,but failed	Yes
6	Normal endometrial	Yes	Yes
12	tissue	Yes	Yes
13		--	Yes
14		--	Yes
17		--	Yes

Table S2. Western blot quantification

Available for download at
https://journals.biologists.com/bio/article-lookup/doi/10.1242/bio.060141#supplementary-data

Biology Open • Supplementary information
